# Supplementary material for: Seeding and transgenic overexpression of alpha‐synuclein triggers dendritic spine pathology in the neocortex
Source: EMBO Mol Med. 2017 Mar 28;9(5):716–31. doi: 10.15252/emmm.201607305 (PMC5412764; doi:10.15252/emmm.201607305)
Supplement: Supplementary file 1 — Appendix [file EMMM-9-716-s001.pdf]

Appendix for

Seeding and transgenic overexpression of α-synuclein triggers dendritic spine pathology in the neocortex

Sonja Blumenstock, Eva F. Rodrigues, Finn Peters, Lidia Blazquez-Llorca, Felix Schmidt, Armin Giese and Jochen Herms*

***To whom correspondence should be addressed**: jochen.herms@med.uni-muenchen.de

Table of contents

Appendix Table S1: Antibodies used in this study 2

Appendix Figure S1. Spine morphology changes in α-syn transgenic mice.. 3
Appendix Figure S2. Distribution of phosphorylated α-syn aggregates in the mouse brain.. ... 4
Appendix Figure S3: Templated misfolding of α-synuclein.. 5
Appendix Figure S4. Presence of microglia in the neocortex.. 6
Appendix Figure S5. Spine morphology changes in PFF-seeded mice (5 mo post-injection). 7
Appendix Figure S6. Presynaptic glutamatergic bouton density does not differ in seeded or transgenic mice.. 8

Appendix Table S1. Antibodies used in this study

Antibody	Source	Host	Dilution
**Mouse α-syn**	New England Biolabs (4179 S)	rabbit monoclonal	1:1000 (WB)
**15G7 human α-syn**	(Neumann *et al*, 2002)	rat monoclonal	1:500 (IF)
**pS129 α-syn**	Abcam (ab59264)	rabbit polyclonal	1:200 (IF)
**Ubiquitin**	Abcam (ab7780)	rabbit polyclonal	1:500 (IF)
**Neurofilament-L (DA2)**	Cell Signaling (2835 S)	mouse monoclonal	1:200 (IF)
**Iba-1**	Wako (019-19741)	rabbit polyclonal	1:500 (IF)
**CD68**	BioRad (MCA 1957)	rat monoclonal	1:1000 (IF)
**VGLUT1**	Millipore (AB5905)	guinea pig polyclonal	1:200 (IF)
**anti-GFP Alexa488**	Invitrogen (A21311)	rabbit polyclonal	1:500 (IF)
**anti-rabbit Alexa 647**	Invitrogen (A21245)	goat polyclonal	1:200 (IF)
**anti-rat Alexa 647**	Invitrogen (A21247)	goat polyclonal	1:200 (IF)

Reference:

1. Neumann, M. *et al.* Misfolded proteinase K–resistant hyperphosphorylated α-synuclein in aged transgenic mice with locomotor deterioration and in human α-synucleinopathies. *J. Clin. Invest.* **110,** 1429–1439 (2002).

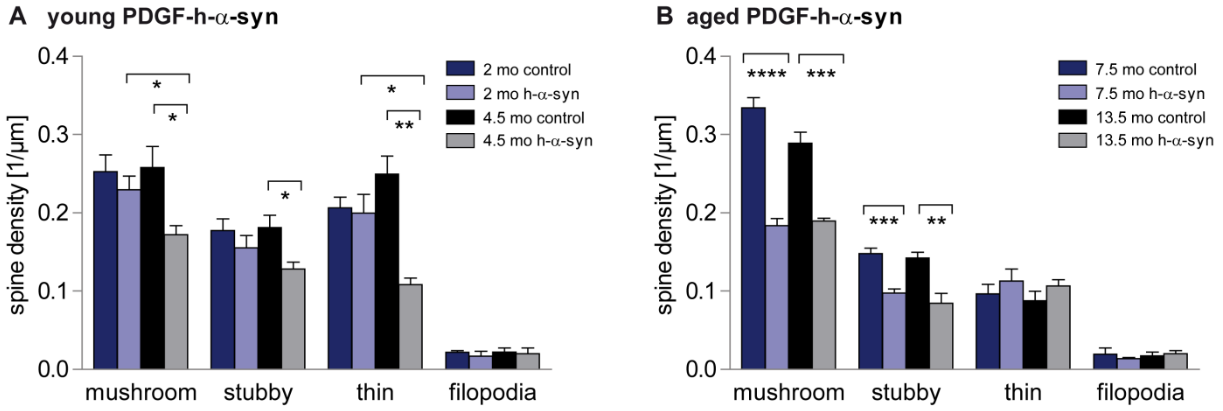

Appendix Figure S1. Spine morphology changes in α-syn transgenic mice. Absolute densities of mushroom, stubby and thin spines in apical tuft dendrites of PDGF-h-a-syn mice compared to controls. (A) In young adult (2 and 4.5 months old) mice, the densites of mushroom ($p_{syn\ 2/4.5mo}$ = 0.0326; $p_{ctrl/syn\ 4.5mo}$ = 0.0255), stubby ($p_{syn\ 2/4.5mo}$ = 0.025) and thin spines ($p_{syn\ 2/4.5mo}$ = 0.0107; $p_{ctrl/syn\ 4.5mo}$ = 0.0012) are decreased. (B) In aged (7.5 and 13.5 months old) mice, the densities of mushroom ($p_{7.5mo}$ < 0.0001; $p_{13.5mo}$ = 0.0005) and stubby spines ($p_{7.5mo}$ = 0.0009; $p_{13.5mo}$ = 0.008) are decreased in h-a-syn mice compared to controls. A: *n* = 3 (control 2 mo), *n* = 4 animals per group. B: *n* = 4 animals per group, mean with s.e.m; *p<0.05, **p<0.01. Student‘s t-test.

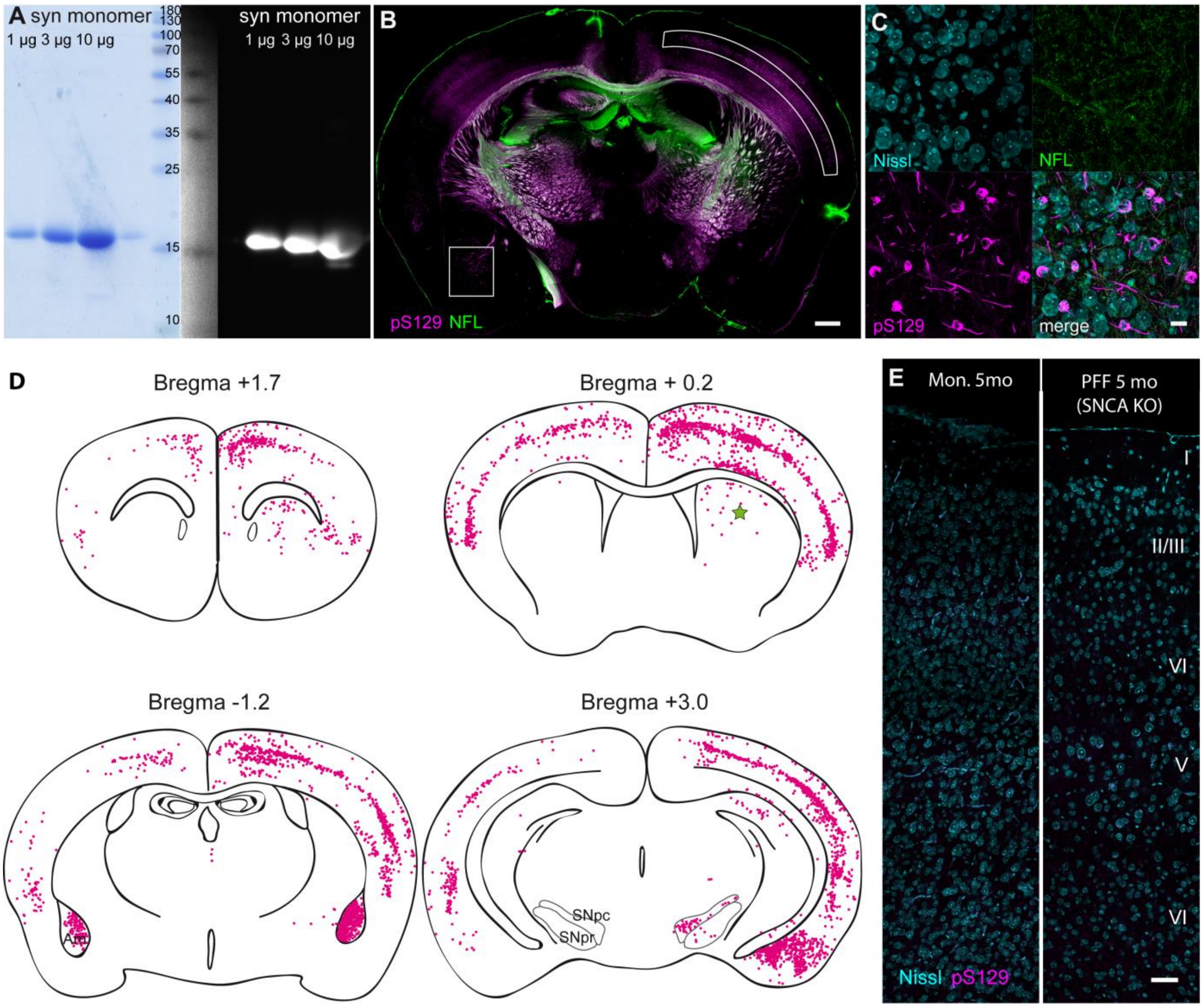

Appendix Figure S2. Distribution of phosphorylated α-syn aggregates in the mouse brain. (A) Full coomassie blot and western blot against mouse α-syn (14 kDa) from the initial momomer preparation of mouse α-syn, demonstrating the purity of the sample before PFF production. (B) 5 months after striatal injection, dense neuronal pS129-immunopositive inclusions are present most prominent in the ipsilateral cortex (layer IV and V) of the injection site and in the amygdala. Due to known cross-reactivity of the pS129 α-synuclein antibody with phosphorylated neurofilament subunit L (NFL), white matter tracts are stained as well (C) Double staining (in the amygdala) distinguishes between false positive staining of white matter and pathological α-synuclein aggregates in cell bodies and neurites. (D) Spread of α-syn aggregates 5 months after seeding. Magenta dots mark the position of intracellular aggregates. Am: amygdala, SNpc: substantia nigra pars compacta, SNpr: substantia nigra pars reticulata. (E) Injection with monomeric α-synuclein into mice expressing endogenous α-syn or PFFs into SNCA KO mice does not lead to the spreading of α-syn aggregates. Image stacks (B,C,E) are depicted as maximum intensity projections. Scale bars: B = 500 µm; C = 20 µm; E = 50 µm.

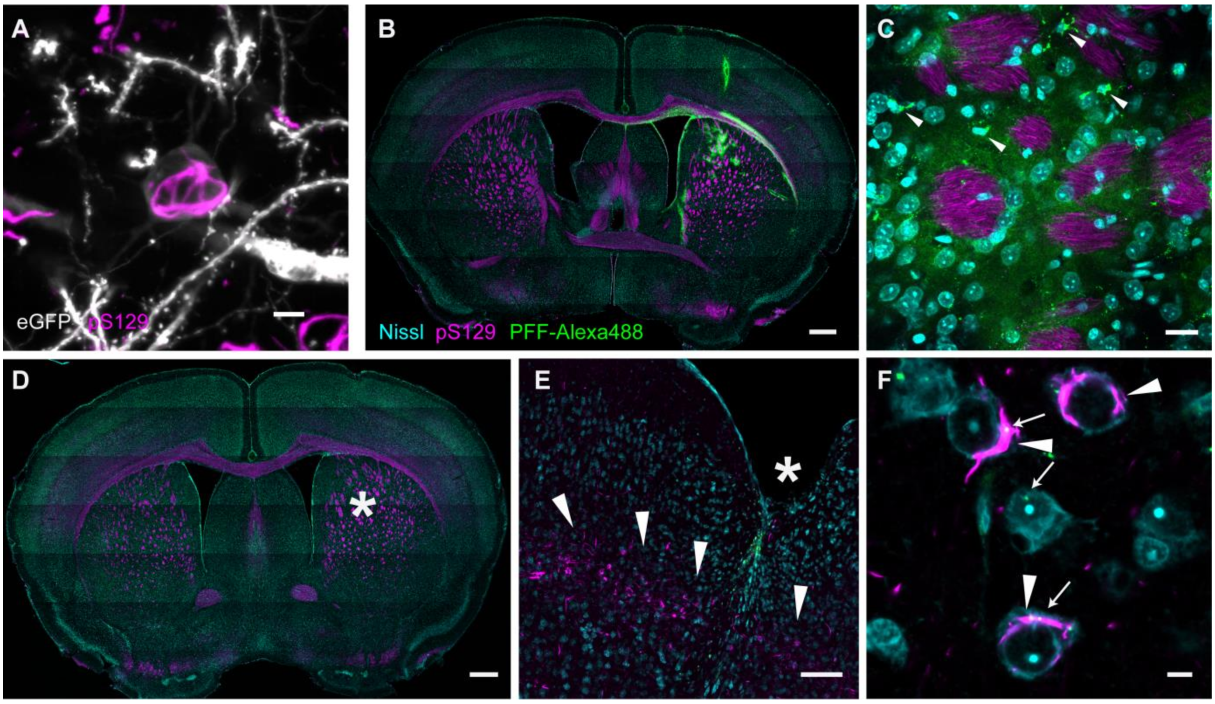

Appendix Figure S3: Templated misfolding of α-synuclein. (A) Neocortical layer V pyramidal neuron containing a phosphorylated α-synuclein inclusion. (B) Brain overview at 1 day post-injection (dpi) with fluorescently labeled PFFs. (C) Injection site in the striatum at 1 dpi. White arrowheads mark cells that have internalized fluorescent PFFs. (D) Brain overview at 6 dpi shows that fluorescently labeled PFFs have been removed from the injection site (asterisk). (E, F) At 30 dpi, small puncta of fluorescent PFFs locate to the injection canal (asterisk) and to some cortical cell bodies (arrows). The developing phospho-α-syn inclusions (arrowheads) however, consist largely of non-fluorescent α-synuclein (F). Scale bars: B,D = 500 μm; E = 100 μm; C = 50 μm; A,F = 5 μm.

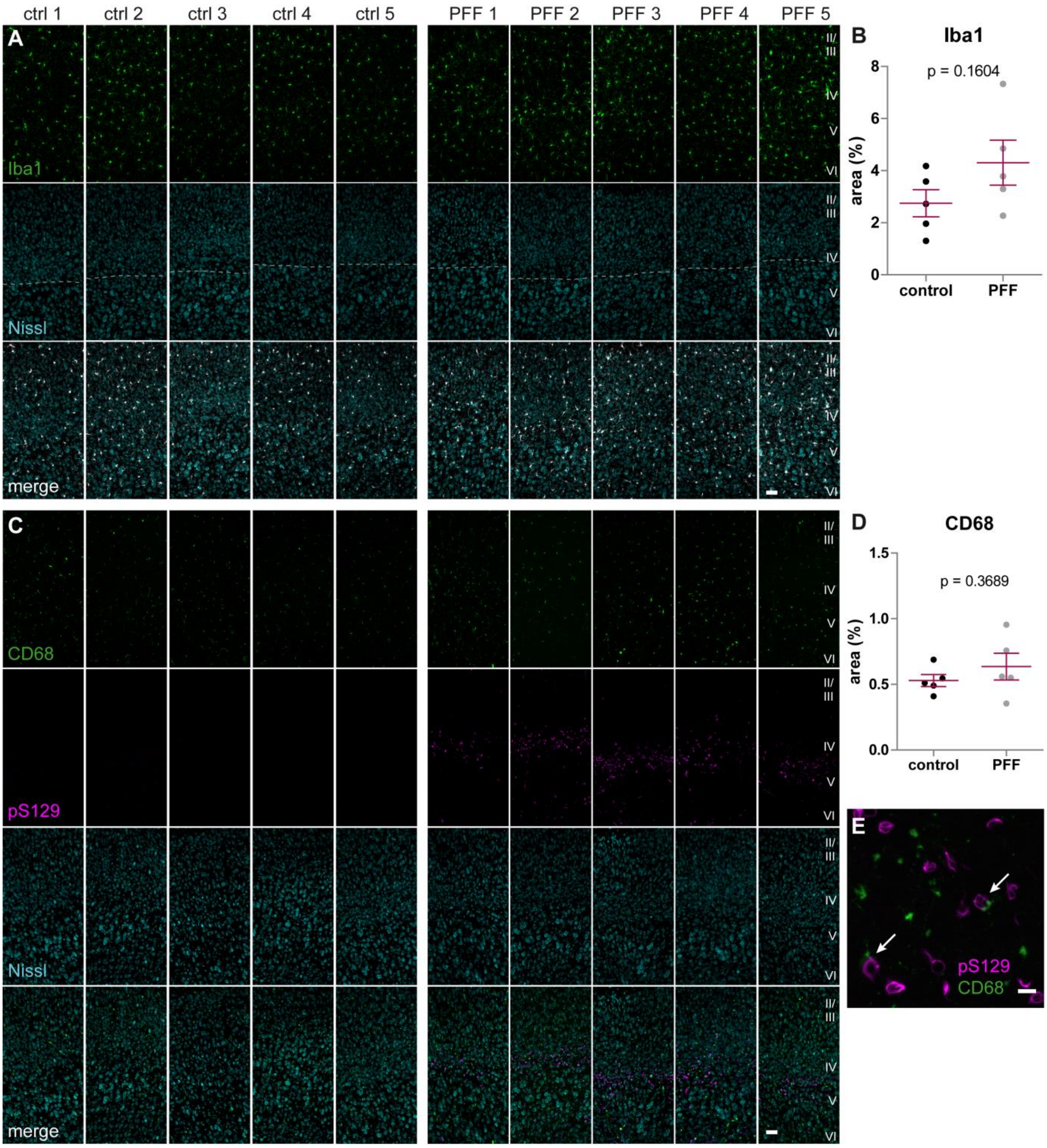

Appendix Figure S4. Presence of microglia in the neocortex. 5 months post-injection with PFFs or PBS (ctrl), staining across cortical layers II-VI were performed against (A) Iba1, marking the total number of microglia (layer IV is indicated as a dashed line, as no double staining with pS129 could be performed) and (C) CD68, marking actively phagocytic microglia. (B,D) area coverage of microglia in % in maximum intensity projections of 10µm of cortical thickness (scaling Z = 2 µm). (E) occasional colocalization of CD68 positive microglia and pS129 positive aggregates (white arrows) is present in layer IV. *n* = 5 animals per group, mean with s.e.m; Student‘s t-test. Scale bars: A,C = 50 µm; E = 10 µm.

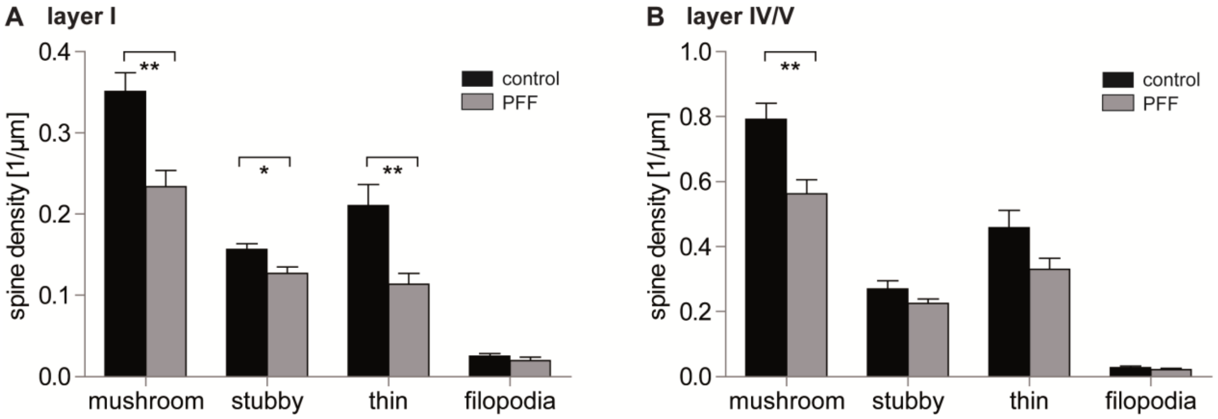

Appendix Figure S5. Spine morphology changes in PFF-seeded mice (5 mo post-injection). Absolute densities of mushroom, stubby and thin spines in (A) apical tuft dendrites of layer I ($p_{mushroom}$= 0.0029, p_{stubby}= 0.0179, p_{thin}= 0.0058) and (B) layer IV/V apical dendrites ($p_{mushroom}$= 0.0046) in PFF seeded mice compared to controls. *n* = 6 (control), *n* = 7 (PFF) animals, mean with s.e.m; *p<0.05, **p<0.01. Student's t-test.

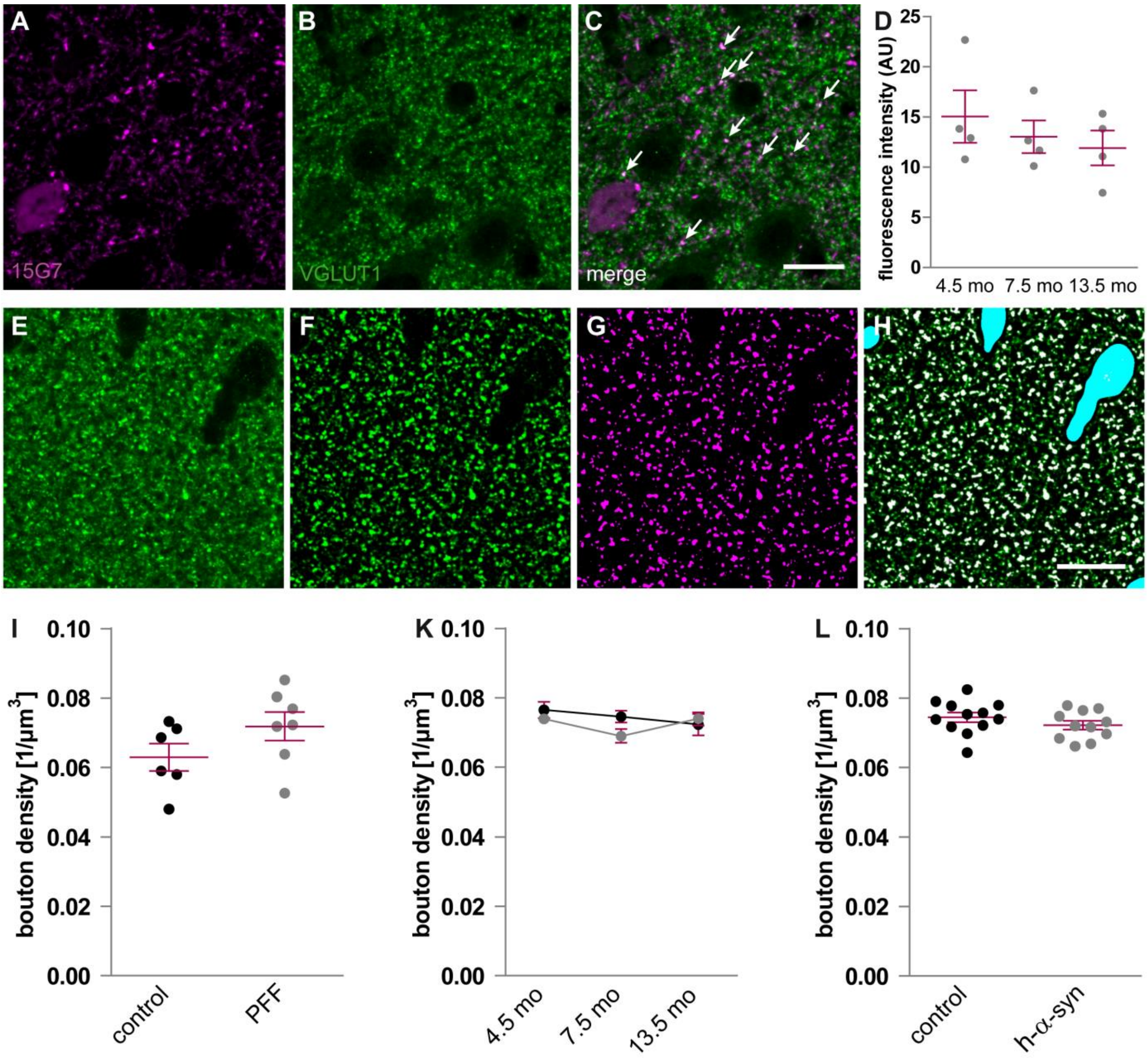

Appendix Figure S6. Presynaptic glutamatergic bouton density does not differ in seeded or transgenic mice. (A) Transgenic α-syn expression and (B) glutamatergic presynapses (C) show overlapping confocal signal. White arrows exemplarily mark double-stained boutons. (D) The expression level of α-syn does not differ significantly across age groups. (E) VGLUT1 immunosignal in green exhibiting a spot-like pattern. (F) The same image is shown after local background subtraction to diminish intensity variations among different stacks and Gaussian filtering to reduce image noise. (G) VGLUT1-positive puncta representing single boutons are shown in magenta as detected by automatic spot detection using Imaris software. (H) Overlay of VGLUT1 immunosignal (green) and the detected boutons (magenta). Cyan color highlights regions that were automatically detected as blood vessels and were excluded from the analyzed volume. (I) The bouton density between control and PFF mice does not differ. n=6-7 animals per group, Mann-Whitney-U-Test, P>0.05. (K) The bouton density does not show a change with age nor between the groups. Two-way analysis of variance "age" $F_{(2)}=1.32$, p>0.05 and "interaction" $F_{(2)}=1.5$, p>0.05; *n* = 4 animals per group. (L) Since age had no effect on bouton density mice of all three age cohorts were pooled and the groups were compared using the unpaired t-test; p > 0.05, n = 12 animals per group, mean with s.e.m. Scale bars: 15 µm.
